# Thermo-Oxidative Stability Evaluation of Bullfrog (*Rana catesbeiana* Shaw) Oil

**DOI:** 10.3390/molecules22040606

**Published:** 2017-04-10

**Authors:** Renata Rutckeviski, Francisco H. Xavier-Júnior, Andreza R.V. Morais, Éverton N. Alencar, Lucas Amaral-Machado, Julieta Genre, Amanda D. Gondim, Eryvaldo S.T. Egito

**Affiliations:** 1 Disperse Systems Laboratory (LaSiD), Pharmacy Department, Federal University of Rio Grande do Norte (UFRN), Av. General Gustavo de Cordeiro-SN-Petropolis, Natal 59010-180, Brazil; renatarut@hotmail.com (R.R.); ffhxjunior@yahoo.com.br (F.H.X.-J.); andrezarochelle@hotmail.com (A.R.V.M.); everton_alencar@hotmail.com (É.N.A.); machado.lucasam@gmail.com (L.A.-M.); jgenre@gmail.com (J.G.); 2 Graduate Program in Health Sciences, LaSiD, UFRN, Av. General Gustavo de Cordeiro-SN-Petrópolis, Natal 59010-180, Brazil; 3 Chemistry Department, Federal University of Rio Grande do Norte, Av. Senador Salgado Filho-3000-Lagoa Nova, Natal 59072-970, Brazil; amandagondim.ufrn@gmail.com

**Keywords:** BO, natural products, fatty acids, physicochemical properties, oxidative stability, thermal stability

## Abstract

Bullfrog oil (BO), a natural product obtained from recycling of adipose tissue from the amphibian *Rana catesbeiana* Shaw, has been recently evaluated as a therapeutic activity ingredient. This work aimed to evaluate the long-term and accelerated thermal oxidative stabilities of this product, which is a promising raw material for emulsion technology development. BO was extracted from amphibian adipose tissue at 70 °C with a yield of 60% ± 0.9%. Its main fatty acid compounds were oleic (30.0%) and eicosapentaenoic (17.6%) acids. Using titration techniques, BO showed peroxide, acid, iodine and saponification indices of 1.92 mEq·O_2_/kg, 2.95 mg·KOH/g oil, 104.2 g I_2_/100 g oil and 171.2 mg·KOH/g oil, respectively. In order to improve the accelerated oxidative stability of BO, synthetic antioxidants butylhydroxytoluene (BHT) and buthylhydroxyanisole (BHA) were used. The addition of BHT increased the oxidation induction time compared to the pure oil, or the oil containing BHA. From the results, the best oil-antioxidant mixture and concentration to increase the oxidative stability and allow the oil to be a stable raw material for formulation purposes was derived.

## 1. Introduction

In the last few years, new biological active products derived from animals have been used as raw material for the food and pharmaceutical industries [1,2]. The bullfrog (*Rana catesbeiana* Shaw) is an amphibian widely used in the meat and leather market [3]. Bullfrog meat has a high nutritional value due to its high content of essential amino acids and low lipid quantity [4]. Normally, the adipose tissue of the bullfrog has been considered a waste product by the food industry, nevertheless it can be reused by biotechnological processing for the production of natural oil, with applications in nutritional, food, cosmetic, and pharmaceutical areas [3].

In Brazilian folk medicine, BO has been regarded as a natural remedy for the treatment of immune and inflammatory diseases [5]. This oil has several advantages for therapeutic applications, including greater biocompatibility and biodegradability, lower toxicity, and a more sustainable process of production when compared to synthetic oils. The versatility of BO for different applications is due to the presence of polyunsaturated fatty acids in its composition, especially the long chain compounds such as eicosapentaenoic acid (EPA) and docosahexaenoic acid (DHA). EPA reduces the risk of cardiovascular diseases, while DHA is essential to the development of the nervous system and vision in mammals [6]. Both components may also act in reducing the synthesis of pro-inflammatory mediators [7], and play an important role in many aspects of health. 

Due to its pharmacologic relevance, formulations containing BO have been studied recently. An emulsion containing this oil was assessed and shown to have increased anti-biofilm activity against different microorganisms [8]. In addition, an optimized BO emulsion for topical use showed promising activity against B16F10 melanoma cells, and no cytotoxicity against 3T3 fibroblast cells [5]. However, the high concentration of polyunsaturated fatty acids can be a problem due to high susceptibility to lipid oxidation, which is the major cause of quality deterioration in many natural products, suggesting a need for stability improvement of the oil prior to formulation use [9].

Autoxidation is the most common oxidation phenomenon that takes place, this occurs through a reaction between oxygen and the unsaturated fatty acids via an auto-catalytic process consisting of a free radical chain mechanism [10]. Physicochemical properties associated with rancidity occur through autoxidation, and changes the taste, color, viscosity, and nutritional quality of the oil, reducing the quality and shelf life of the oil [11]. In general, oxidative reactions, which are dependent on temperature, storage time and chemical characteristics [12], are induced by the presence of initiators such as heat, free radicals, light and metal ions [13]. In order to delay or to prevent oxidation, synthetic antioxidants, such as BHT and BHA have widespread use as additives for food, pharmaceutical, and cosmetic products. These antioxidants are able to donate hydrogen to free radicals during the initial stages of the oxidation reaction, preventing autoxidation [14]. Natural antioxidants from isolated plant extracts (such as ascorbic acid, tocopherols, carnosic acid, and rutin) are also used to prevent oxidation. However, synthetic antioxidants are safe, effective, cheaper, and easier to process than natural antioxidants. Thus, the aim of this work was to evaluate the effect of synthetic antioxidants on the formation of primary products of oxidation from BO, as a preliminary quality control measure for this animal product for the purpose of developing new oil based formulations. In this study, extraction, physicochemical and chemical characterizations of BO were performed. In addition, the thermo-oxidative stability of pure BO was compared to BO containing added antioxidants (BHA and BHT), as a study for storing oil samples at room temperature and under thermal stress.

## 2. Results and Discussion

### 2.1. Extraction and Physicochemical Characterization of Bullfrog Oil

The extraction process of the BO from adipose tissue using a mild temperature extraction of 70 °C for 30 min presented a yield of 60.0% ± 0.9%. The residue obtained after the extraction process was composed of tissue remains, water, and a whitish layer. This result corroborated to that found by Mendez et al. (1998) who reported a yield of 61.7% after extraction at 98 °C for 30 min. Lopes et al. (2010) and Machado et al. (2016) obtained a yield of 74% and 74.8%, respectively, using a different temperature (80 °C) for 40 min. In this work, the mild temperature extraction had a lower yield than other solvent extraction systems (i.e., the Soxhlet and Folch methods). The hot extraction method is advantageous due its low cost, time involved, and the absence of chemical residue that can cause negative impacts on human health and on the environment [15]. Furthermore, added solvents reduce the physicochemical quality of the oil, due to increased peroxide levels [5]. In addition, it was reported that the unsaturated fatty acid proportion an increased with an increase in temperature up to certain limits [16].

The chemical composition of the fatty acids containing in the BO after derivatization with BSTFA (*N*,*O*-bis(trimethylsilyl)trifluoroacetamide) reagent was obtained by gas chromatography-mass spectrometry (GC/MS), considering components with a relative peak area greater than 0.7%. A list of those components from the extracted BO included 10 components corresponding to 83.9% of all compounds identified (Table 1A). Among the fatty acids, eicosapentaenoic acid (EPA, 20:5 *n*−3) was the most abundant (17.6%), while oleic acid (18:1 *n*−9) (30%) was the most abundant unsaturated fatty acid. Regarding other studies involving animal oils, unsaturated fatty acids are present at higher concentrations than saturated fatty acids in fish oils [3]. The major content of polyunsaturated fatty acid was derived from the omega family compounds. In addition, the content of EPA was similar to that found by Machado et al. (2016) for BO (17.6%), and Bimbo (1998) for sardine oils (16.0%) and anchovy oil (22.0%) [17]. On the other hand, the EPA content found for BO by Mendez (1.5%) [16] and Silva (1999) (0.46%) were lower than the amount found on this work. This increased amount of EPA may be associated with the extraction method used, or with the bullfrog’s diet, sex, age, and acclimation to low temperatures, which leads an accumulation of polyunsaturated fatty acids [7,18]. Indeed, long-chain polyunsaturated fatty acids such as EPA, maintain the structure of cell membranes, and are essential in the synthesis of intracellular lipids in the epidermis corneous layer.

**Figure 1:**
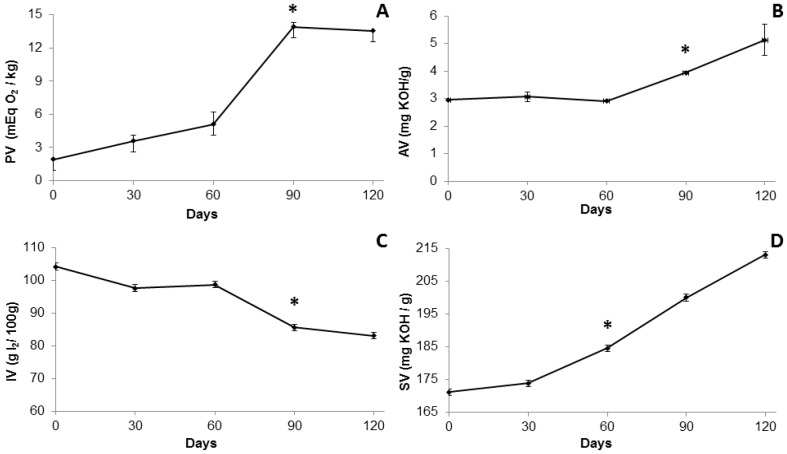
Physicochemical properties of the BO stored at room temperature over 120 days. (**A**) Peroxide value; (**B**) Acid value; (**C**) Iodine value; (**D**) Saponification value. * *p* ˂ 0.05.

Figure 1 shows the physicochemical properties of BO stored at room temperature over 120 days. Due to the lack of standardization for the BO, the results were compared to the values for fish oils. The formation of peroxide over time was important to identify the stage of the primary products of oil oxidation. Immediately after extraction, the peroxide value (PV) (1.93 ± 0.02 mEq O_2_/kg oil) of the BO (Figure 1A) was within the limits specified in the literature for fish oils (≤5 mEq/kg) [19]. After 60 days, the peroxide formation rate increased sharply due to increased degradation of unsaturated fatty acids by auto-oxidation. Between 90 and 120 days, the peroxide formation rate decreased, perhaps due to its decomposition and the formation of secondary oxidation products, such as ketones and aldehydes, indicating a later stage of oil oxidation and rancidity [20].

The acid value (AV) indicates oil conservation and quality of the adipose tissue, as well as the ideal conditions for extraction method and storage. The AV immediately after extraction was 2.95 ± 0.01 mg·KOH/g oil (Figure 1B), indicating that the hydrolysis reactions occurred during adipose tissue storage or oil extraction. However, this value remained constant for up to 60 days at room temperature, and within the limits (≤3 mg·KOH/g) established by the Codex Alimentarius [19], showing good oil conservation status during this period. After 60 days, AV increased significantly, indicating triglyceride hydrolysis reactions, decreasing oil neutralization due to the presence of free fatty acids (FFAs). High content of FFAs in the oil reduces its commercial value, due to the increase in acidity and the reduction of oil quality [21]. After 60 days of storage at room temperature, this oil showed high AV, which may be reduced through oil refining, addition of antioxidants, or better storage conditions for both the adipose tissue and the oil [22].

The iodine value (IV) is a measure of the oils’ unsaturation, which is based on the ability of an unsaturated carbon-to-carbon bond to react with halogen atoms (iodine). The IV obtained for BO was 104.21 ± 1.0 g I_2_/100 g oil, showing a greater presence of unsaturation than crude fish oil from *Nile tilapia* (84.5 g I_2_/100 g oil) [11]. According to Figure 1C, during 60 days of storage, the IV decreased by 5%, remaining within the allowed value for most fish oils. Between 60 and 90 days, IV decreased by 16%, due to a greater reduction in the number of unsaturation points as a result of oxidation or breakdown of long chain fatty acids [23]. 

The saponification value (SV) (Figure 1D) is useful for predicting the chain length of fatty acids, wherein a higher SV indicates a shorter chain length. Oils with high saponification values are important in the food and cosmetic industry, because they are less susceptible to deterioration in comparison to long fatty acids chains [24]. The SV (171.12 ± 0.97 mg·OH/g oil) obtained after extraction was lower than the value reported for *Nile tilapia* crude fish oil (194.91 mg·KOH/g oil) [11]. However, after 60 days, the SV showed a significant increase, which may be due to the greater amount of short chain fatty acids, indicating that the level of long-chain fatty acids decreased over time [23]. 

The organoleptic properties of BO were evaluated over 120 days at room temperature and at 60 ± 2 °C for 15 days. A conventional sensory profile was established for the odor and color of the BO. Immediately after extraction, this oil presented a light yellow color, transparency and characteristic aroma, which was maintained for up to 90 days of storage. After 90 days, changes in color were observed, as well as an increase in a rancid smell, which could be related to the decrease in the PV (Figure 1A) [12]. This decrease can indicate hydroperoxide decomposition to aldehydes and ketones, which produces a characteristic odor in oxidized oils. [25]. With regard to BO samples in accelerated stability, samples of BO and BO with BHA at minimum concentration (BOAm) showed a rancid odor and a moderate yellow color after 15 days of storage. However, the other samples remained unchanged. Therefore, the very moderate change in the sensory profile was reflected in the chemical lipid oxidation parameters [26].

Oils have specific refraction index values, thus, a variation from the normal value indicates adulteration or sample degradation [21]. In this study, the refractive index value was 1.4701, which is equal to the value reported for Sorubim crude fish oil [11]. Additionally, this refraction index was close to the range reported in the literature for some fish and vegetable oils [27]. 

### 2.2. Accelerated Oxidative Stability of Bullfrog Oil

#### 2.2.1. Physicochemical Changes

**Figure 2:**
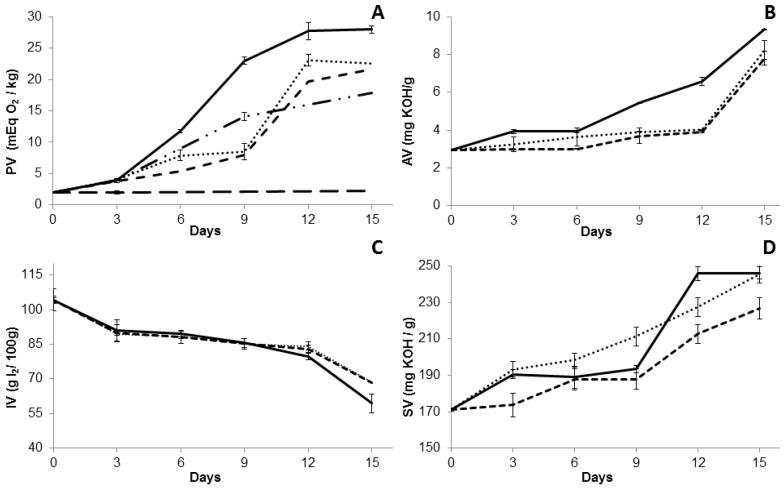
Physicochemical properties of bullfrog oil from the Schaal oven test conducted over 15 days at 60 °C. (**A**) Peroxide value; (**B**) Acid value; (**C**) Iodine value; (**D**) Saponification value. Samples: BO (**―**); BOAm (∙∙∙∙); BOAmx (**—**∙∙); BOTm (----); BOTmx (**– –**).

Accelerated oxidative stability testing was performed with pure BO and BO with addition of butylhydroxytoluene (BHA) or buthylhydroxyanisole (BHT) antioxidants at the minimum (BOAm and BOTm) and maximum (BOAmx and BOTmx) concentrations. All BO samples (BO, BOTm, BOTmx, BOAm, BOAmx) containing different concentrations and types of antioxidants, were stored at 60 ± 2 °C for 15 days. The Schaal oven test is an accelerated stability method which has a good correlation with the assessment carried in oils stored at room temperature [28]. Figure 2 shows the physicochemical parameters evaluated for the BO with and without antioxidants under the Schaal oven test. The primary products of lipid oxidation, as indicated by the PV (Figure 2A), increased with the time of exposure to heat for most samples. The PV of the BO started to increase from the third day, reaching a maximum value of 28.85 ± 0.5 mEq·O_2_/kg oil after 15 days of storage at 60 ± 2 °C. Due to a lack of parameters for the PV, significant differences were found for this index compared to other oils subject to heat treatment [29]. These differences can be attributed to variations in the composition of the oils. Oils with a high amount of unsaturated fatty acids are more susceptible to oxidation, and a higher rate of peroxide production. The addition of low levels of antioxidants resulted in a decrease of PV compared to the pure oil. BHT at 0.02% rather than BHA at 0.02%, provided better protection against oil deterioration. Moreover, after 15 days, the addition of BHA at 0.5% to the BO reduced the PV two-fold, while adding BHT at 0.5% resulted in a significant PV reduction of 13-fold compared to the pure oil. The formation of peroxides in the BO and BOAm samples decreased after 12 days of storage, which may be attributed to the last step of reaction, resulting in the peroxide decomposition and formation of secondary oxidation products [12]. The difference in the oxidation rate profile may be associated to the presence of alkyl groups into 6-positions in the BHT, which increases the electron density on the hydroxyl group by an inductive effect and, thus, increases the hydrogen donation ability. Besides the chemical structure of BHT, the concentration of BHT influenced both the levels of peroxides formed, and the oxidative stability of the oil. This may partly explain why BHT was more effective as an antioxidant compared to BHA in the BO samples [30]. 

Figure 2B shows the AV of the BO under the Schaal oven test. The AV of the BO sample increased 1.3-fold over six days, reaching a value of 3.93 mg·KOH/g of oil. From this time, the AV increased rapidly (3.2-fold), reaching 9.33 mg·KOH/g oil after 15 days of storage. This difference may be due to the increased of hydrolysis of the triacylglycerides, which increase the acidity of the oil, indicating advanced stages of degradation [31]. In all samples, the AV increases were lower than the oil without antioxidant. Figure 2C shows a reduction of the 43% in the IV (BO sample) after 15 days of the Schaal oven test, indicating sample degradation and a decrease in double bonds. However, the addition of both BHT and BHA at 0.02% showed a reduction of approximately 33% in the IV. As shown in Figure 2D, after 15 days of storage there was an increase in the SV of the samples (BO, BOAm and BOTm), which indicates that the average molecular weight of the fatty acids decreased over time under heating. The BO and BOAm samples showed greater saponification values compared to BOTm, indicating that the addition of BHT was able to maintain fatty acid chain size.

**Figure 3:**
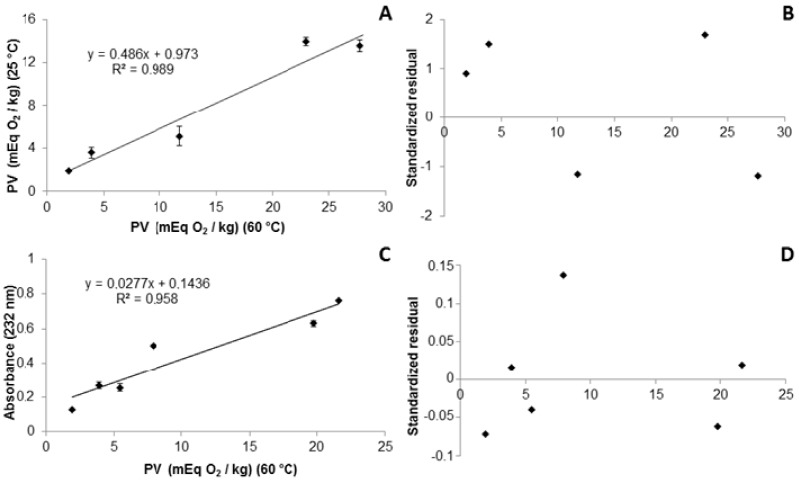
Correspondence between the peroxide values (PV) of pure bullfrog oil obtained from the Schaal Oven test at 60 ± 2 °C over 15 days, and at room temperature over 120 days (**A**); and their respective standardized residual (**B**); Correspondence between the peroxide values (PV) and the UV-vis spectrum absorbance at 232 nm of the bullfrog oil containing BHT 0.5% obtained in the Schaal Oven test at 60 °C (**C**); and its respective standardized residual (**D**).

A satisfactory correlation coefficient (R = 0.989) was obtained for the formation of peroxides during the oxidation of BO at room temperature over 120 days, and at 60 °C for 15 days (Figure 3A). The model’s goodness of fit was checked by the coefficient of determination R^2^ = 0.979, showing that 97.9% of the variability in the response could be explained by the model. According to the ANOVA regression model, the linear model was significant (*p* < 0.05). This was evidenced by the Fisher’s test, which provided a model F-value (F model = 190.9) that was much greater than the tabulated F-value (F Tab = 7.7) at the 5% level, indicating a high degree of adequacy of the linear model. This predictive model presented homoscedasticity in the residues distribution (Figure 3B), indicating that the variance of the error was maintained throughout the observations.

#### 2.2.2. Conjugated Dienes and Trienes

**Figure 4:**
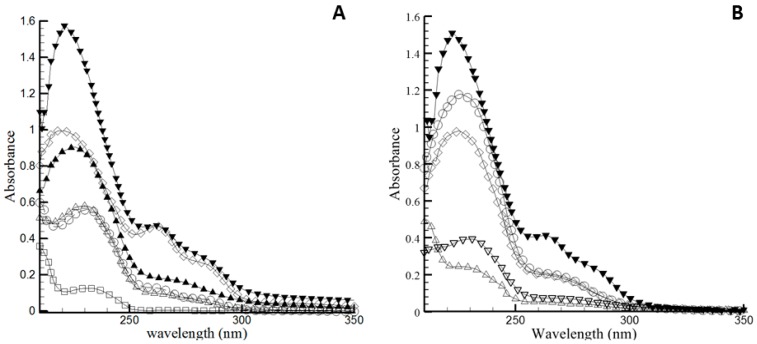
UV-visible absorption spectra of bullfrog oil exposed to the Schaal oven test at 60 °C. (**A**) Represents the spectra of pure bullfrog oil (BO): (□) 0 day, (ο) 3 days, (∆) 6 days, (▲) 9 days, (◊) 12 days, (▼) 15 days. (**B**) corresponds to different BO samples containing antioxidants exposed to the Schaal oven test after 15 days: (▼) BO, (ο) BOAm, (◊) BOTm, (∇) BOAmx and (∆) BOTmx.

Conjugated dienes (CDs) are primary oxidation products that contain double bonds that alternate with a single bond, and show an absorption in the UV region at 232 nm. Conjugated trienes (CTs) contain three alternating double bonds and UV absorption occurring at 273 nm. Figure 4 shows that the CD and CT values for pure BO increased progressively with heat exposure time. Pure oil showed a low initial concentration of CD of 6.5 ± 0.2, which may be related to the presence of initial oxidation products (peroxides). After 15 days of storage, both CD and CT values increased. The increase in CT value is due to the instability of peroxides under heating, leading to secondary oxidation products that absorb at 273 nm, indicating advanced oxidation over time. During this process, a displacement of the position absorbance band occurs, due to the progressive thermal oxidation of the oil, resulting in the stabilization of the radical formed during oxidation, by the rearrangement of the polyunsaturated lipid double bonds [32]. 

On the other hand, the addition of antioxidants BHT and BHA reduced the intensity of the absorption bands at both 232 nm and 273 nm after storage at 60 °C over 15 days (Figure 4B). The CD value after addition of BHA at concentration of 0.02% and 0.5%, was reduced by 22% and 68%, respectively. However, addition of BHT at 0.02% and 0.5% reduced the CD value by 40% and 83%, respectively. It is also noteworthy that the addition of BHT at 0.5% (BOTmx) was able to maintain the CD value at low levels over 15 days of storage, corroborating with the PV results. CT showed a significant reduction with the addition of BHT at both concentrations. Therefore, it is possible to affirm that the BHT antioxidant is effective at increasing the oxidative stability of BO at both concentrations tested. 

A good correlation of peroxide formation between the CD and the PV analyses was observed with BHTmx exposed to the Shaal Oven test at 60 °C over 15 days (Figure 3C). The model’s goodness of fit was checked by the coefficient of determination R^2^ = 0.958, indicating that 95.8% of the variability in the response could be explained by the model. According to the ANOVA regression model, a significant linear model (*p* < 0.05) and a homoscedastic distribution of the residue was observed (Figure 3D). These satisfactory correlations occurred due to the initial stages of the autoxidation of the polyunsaturated fatty acids, where the PVs are increased concomitantly with an increase of absorption at 232 nm, in relation to the respective CD.

#### 2.2.3. Rancimat^®^

The Rancimat^®^ method allows the oxidative stability of oils to be analyzed by determining the induction time (IT). This analysis characterizes the ability of the oil to resist to the oxidation process and represents an important parameter to identify the quality of the oil. The BO sample showed an IT of 0.32 ± 0.01 h (Table 1B), which was lower than the result reported in the literature (1.7 h) [16]. Indeed, Bakry et al (2016) and Almeida et al (2015) have obtained an IT of 0.16 h and 1.6 h for tuna oil and fish oil, respectively. The differences in the IT may be associated with the higher amount of polyunsaturated fatty acids in the BO that provides a lower melting point, increasing susceptibility to oxidation and, consequently, a decrease in IT [27]. The addition of both BHA at 0.02% or 0.5% decreased the IT to 0.05 h, compared to the pure oil. The addition 0.02% of BHT increased the value of IT by two-fold (0.72 ± 0.02 h), whereas the addition of the BHT at 0.5% significantly increased the IT value 24 times (7.88 ± 0.13 h) in comparison to the pure oil (Table 1B). Therefore, the antiradical activity of BHT in the BO depends on both its chemical structure and its concentration [33,34].

#### 2.2.4. Fourier Transform Infrared Spectroscopy Analysis 

**Figure 5:**
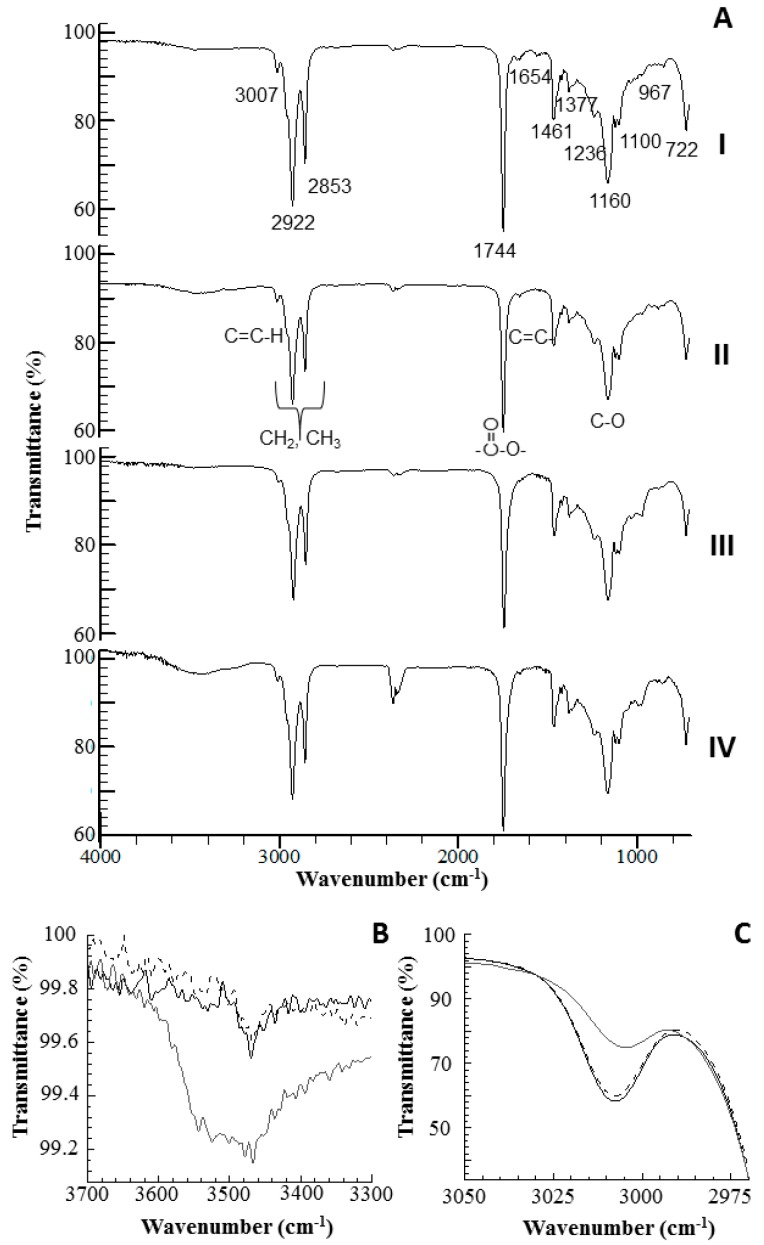
(**A**) Important changes in the Fourier transform infrared spectroscopy spectra of the bullfrog oil without antioxidants (BO), under oxidative conditions (60 °C), (I) 0 day, (II) 6 days, (III) 12 days, (IV) 15 days. B and C represent the changes in BO containing antioxidants after 15 days: (**B**) formation of hydroperoxides near 3445 cm^−1^, (**C**) reduction of the double unconjugated around 3007 cm^−1^. (**---**) BO, (**—**) BOTmx and (**—**) BOAmx.

Fourier transform infrared spectroscopy (FTIR) has been proven to be an important tool in assessing the quality and composition of oils during oxidation [35]. Figure 5 shows the spectra of pure BO at 60 °C for 15 days. The fresh BO presented a prominent band of carbonyl stretching near 1743 cm^−1^ and C-O bond vibrations in 1236, 1160 and 1100 cm^−1^, which express the ester linkage in triacylglycerol (Figure 5-AI). The low intensity band at 3007 cm^−1^ is attributed to the C-H stretching vibrations at the cis double bonds (=CH) of the polyunsaturated fatty acids. This assignment is supported by the deformation of the CH outside the plane of the band 722 cm^−1^, and also by the cis structure (-CH=CH-) stretching vibration at 1654 cm^−1^.The band near 1461 and 1377 cm^−1^ may be assigned to the bending vibrations of the aliphatic CH_2_ and CH_3_ groups [36]. FTIR changes in the spectra of BO during the oxidation process at six, 12 and 15 days are shown in Figure 5A (II, III and IV, respectively).

The BO sample analyzed by the Schaal oven test showed a small decrease in intensity and frequency band near 3007 cm^−1^ after 15 days, which can be attributed to the loss of the double bonds in the unsaturated fatty acids. During the oxidation process, these acids promoted the formation of hydroperoxides (primary oxidation products), which showed a band near 3445 cm^−1^, reaching their maximum intensity after 12 days of heating [35,37]. However, after 15 days, this band was almost undetectable, due to decomposition of the hydroperoxides [38]. The spectral region of 1600–1800 cm^−1^ provides information about secondary oxidation products, especially aldehydes, which are responsible for rancidity. The band located near 1744 cm^−1^ showed a broadening and a slight decrease in frequency after 15 days of storage, indicating the slight presence of aldehydes [35,38]. 

FTIR of oil samples with 0.5% BHT showed less of a change when compared to the pure oil and to the 0.5% BHA sample. Among the regions used to assess the degradation of oils, the band around 3445 cm^−1^ increased in intensity, due to increased formation of hydroperoxides in the presence of BHA (Figure 5B). These hydroperoxides are formed due to the oxidation of unsaturated fatty acids, reducing the cis double bonds (Figure 5C).

#### 2.2.5. Thermal and Oxidative Stability

**Figure 6:**
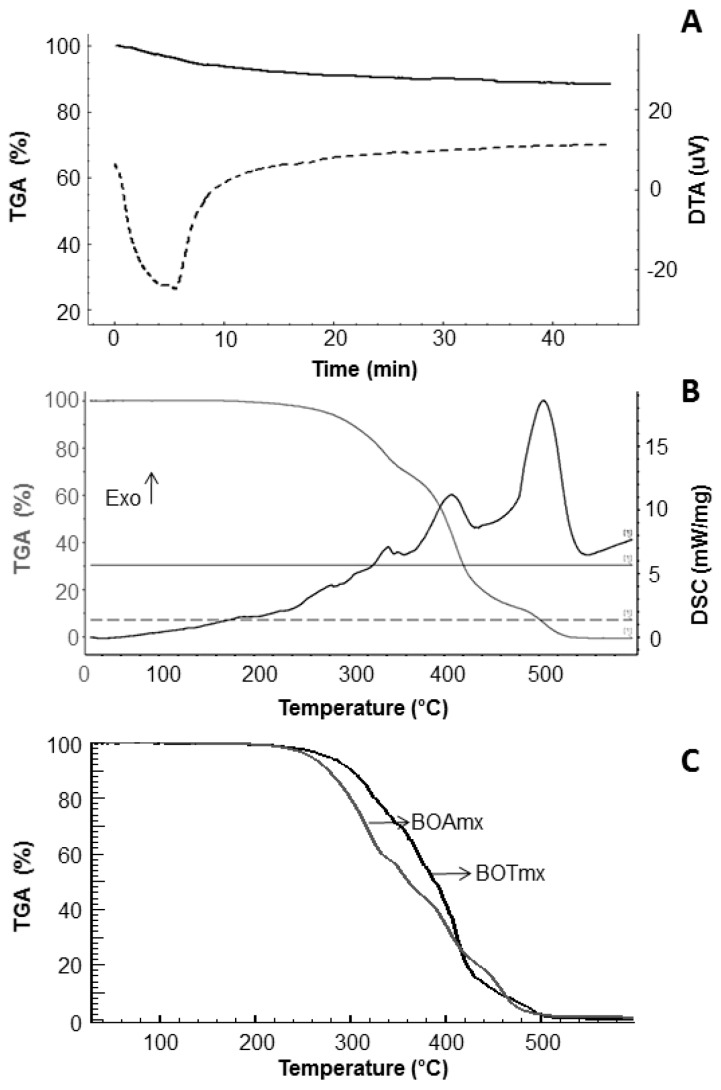
Thermal analysis of BO samples. (**A**) Thermogravimetric analysis—TGA (**—**) and differential thermal analysis—DTA (**---**) curves of fatty tissue of the bullfrog by the isotherm method; (**B**) TGA (**—**) and differential scanning calorimetry—DSC (**—**) curves of bullfrog oil without antioxidants; (**C**) TGA curve of bullfrog oil containing antioxidants at the maximum concentration BOTmx (**—**) and BOAmx (**—**).

The study of thermal stability is important to verify if the oil maintains its original properties at high temperatures. Thermal stability depends on the oil chemical structure and the stability changes according to the source from which the oil was obtained. Thermogravimetric analysis (TGA) and differential thermal analysis (DTA) curves for the adipose tissue of the BO are shown in Figure 6A. It observed that after 10 min of heating at 70 °C, an endothermic event with 6% of weight loss occurred, due to the disruption of fat cells, and the onset of oil release. After 10 min, there was a weight loss of 5%. These results showed that the parameters chosen for oil extraction did not negatively affect its overall quality.

From the thermal behavior expressed by the TGA curve (Figure 6B), it was observed that the BO remained stable at 200 °C, after three significant stages of mass loss. The first degradation event occurred from 200 to 350 °C, with a weight loss of 28%, attributed to the decomposition of the polyunsaturated fatty acids. This event did not show a well-defined peak, which may be due to the presence of impurities such as phospholipids, free fatty acids and other materials presented in the crude oil [12]. The second event (350 to 450 °C), with 56% mass loss, is related to the degradation of unsaturated and saturated fatty acids; the formation of cyclic monomers, dimers and biopolymers also occurred at this stage [31]. The last event (450 to 530 °C) is related to the decomposition products formed in the previous stages, with a 16% mass loss. By differential scanning calorimetry (DSC) analyses, it was observed that the three successive exothermic events, with the first two located during the higher weight loss period, related to the thermal oxidation or the oxidative thermal degradation of the oil. The last event, between 450 to 530 °C, was caused by the release of the energy from the total decomposition of the oil. In addition, the presence of BHT in BO promotes the increase of its thermal stability, as characterized by the displacement of the thermogram profile (Figure 6C).

## 3. Materials and Methods

### 3.1. Materials 

Bullfrog adipose tissue was provided by Asmarana Produtos Naturais (Natal, RN, Brazil). Isooctane and BSTFA was purchased from Sigma Aldrich Inc (St. Louis, MO, USA). Sodium hydroxide, *n*-hexane, potassium hydroxide, diethyl ether, hydrochloric acid, sodium thiosulfate and sodium carbonate were acquired from VETEC (Rio de Janeiro, RJ, Brazil). Butylhyldroxytoluene, buthylhydroxyanisole, potassium biphthalate, potassium dichromate and Wijs solution were purchased from Labsynth (São Paulo, SP, Brazil). Acetic acid, absolute ethyl alcohol, sodium bicarbonate, potassium iodide, starch and chloroform were acquired from Isofar (Rio de Janeiro, RJ, Brazil). Phenolphthalein was provided from Biotec Chemicals (Londrina, PA, Brazil). Ultrapure water was obtained from a Millipore purification system (Milli-Q plus^®^, Millipore, St Quentin en Yvelines, France). All chemicals were of reagent grade and used as received.

### 3.2. Bullfrog Oil Extraction

BO samples were extracted from the adipose tissue of the amphibian. The adipose tissue was triturated in a blender at 4000 rpm for one minute. The fatty material was heated on a hot plate at a controlled temperature of 70 ± 1 °C for 30 min under stirring at 300 rpm (IKA^®^, RH basic model KT/C, Staufen, Germany). The sample was filtered through a filter paper (pore size approximately 26 µm) to remove the large residues. The extracted oil was dried with sodium sulfate, centrifuged at 8792 g for 15 min (Kubota KR-20000T, Rotor RA-1M, Tokyo, Japan) to remove insoluble material, and the supernatant was filtered through a 0.22 µm membrane filter (Millex^®^, Millipore, France). The BO was stored in an amber borosilicate glass in a closed oven without light, at 25 ± 2 °C for 120 days, or at 60 ± 2 °C for 15 days.

### 3.3. Physicochemical Characterization of Bullfrog Oils 

#### 3.3.1. Peroxide, Acid, Iodine and Saponification Values

Physicochemical analyses were performed according to the methods proposed by the United States Pharmacopeia (USP 30) [39], and the American Oil Chemists Society [40], in order to establish a control quality profile for BO stored at 25 ± 2 °C for 120 days. PV was determined using a saturated solution of potassium iodide and starch solution, with subsequent titration of the samples with sodium thiosulfate (0.01 N). The AV analysis was performed by titrating the samples with sodium hydroxide (0.1 N) and using phenolphthalein as an indicator. IV was determined by titrating Wijs solution and sodium thiosulfate (0.1 N) using a starch indicator. SV was assessed using a reflux condenser with subsequent titration of the samples with hydrochloric acid (0.5 N) and alcoholic phenolphthalein as indicator. These analyses were performed at 25 ± 2 °C over 120 days.

#### 3.3.2. Organoleptic Characteristics

Appearance and color of the samples were visually characterized, while the odor was analyzed by an evaluator trained in sensory analysis specific for BO. The criteria described below were established by the evaluator to determine organoleptic characteristics. A conventional sensory profile was established for the BO odor and color attributes. Three milliliters of BO were placed into an odorless test tube of 5 mL capacity, analyzed in duplicates, and then compared against a fresh BO sample. The evaluator gently swirled the test tube and posteriorly sniffed the headspace of the sample. The test tube was then closed quickly [26,41]. 

#### 3.3.3. Refractive Index

The samples stored at room temperature had their refractive index measured at 20 °C using a digital refractometer Abbe (Quimis, Diadema, SP, Brazil). Oil samples were analyzed immediately after extraction.

### 3.4. Chemical Characterization of Bullfrog Oil

The identification of BO constituents was performed by GC-MS using a Gas-Chromatography Ultra equipped with an ITQ Tune mass selective detector (Thermo Scientific, Waltham, MA, USA). A fused silica capillary column (25 m × 0.32 mm i.d., 0.5 µm) film thickness coated with cross-linked 5% phenyl polysilphenylene-siloxane (SGE Analytical Science Pty Ltd., Melbourne, Victoria, Australia) was used. The GC–MS injector was set at 250 °C and the column was set at 90 °C with a heating ramp at 2 °C·min^−1^ to 150 °C, then isothermal heating at 20 °C·min^−1^ to 300 °C. Helium was used as the carrier gas, at a flow of 1 mL·min^−1^. The GC–MS electron ionization system was set at 70 eV. Samples were derivatizated with BSTFA reagent and the volume injected was 1 µL. The BO components were identified by comparing their mass spectra with data obtained from electronic libraries (MAINLIB, WILEY6, TOX.HP, Acides.hp, SAMM, RTLPEST3.HP and pmw.tox2). 

### 3.5. Oxidative Stability of the Bullfrog Oil 

#### 3.5.1. Preparation of Bullfrog Oil Samples

BO without antioxidants (BO), BO with BHT at the concentration of 0.02% (BOTm) and 0.5% (BOTmx), and BO with BHA at concentrations of 0.02% (BOAm) and 0.5% (BOAmx), were used during this study. Thirty grams of each oil sample (pure or with antioxidant added) were placed in 50 mL glass bottles. Accelerated stability studies were performed under Schaal oven test conditions (60 ± 2 °C) over 15 days. The accelerated oxidative stability of the five different BO preparations (BO, BOTm, BOTmx, BOAm, BOAmx) were monitored by physicochemical characterization as described above, by CD and CT, IT (Rancimat^®^), and FTIS. 

#### 3.5.2. Conjugated Dienes and Trienes

The presence of CD and CT conjugates formed during the oxidation process was determined by the Evolution 60S UV–Vis Spectrophotometer (Thermo Scientific, Waltham, MA, USA) in the scan interval of 200–500 nm at 25 ± 2 °C. The BO samples (BO, BOTm, BOTmx, BOAm, BOAmx) were stored at 60 ± 2 °C for 15 days. 1% of the BO samples were diluted in hexane and vigorously stirred for one minute prior to analysis. Absorbance at 232 and 273 nm were used for the identification of CD and CT, respectively. The absorbance was determined against a blank made from hexane in both wavelengths. CD and CT values from their respective absorbances were expressed as extinction values (E^1%^_1cm_) using the Equation (1):

E^1%^_1cm_ = A_λ_/(*C_L_* × *l*) (1)

where E^1%^_1cm_ was the extinction value, A_λ_ was the absorbance measured at either 232 nm (for CDs) or 273 nm (for CTs), *C_L_* represented the concentration of the lipid solution in g/100 mL, and *l* was the path length of the cuvette in cm [42].

#### 3.5.3. Rancimat^®^

IT was evaluated by the Rancimat^®^ (Metrohm model 873, Herisau, Switzerland). Three grams of each oil sample (BO, BOTm, BOTmx, BOAm, BOAmx) were heated at 110 ± 1 °C under airflow of 10 L/h. The volatile compounds produced during oxidation were captured and analyzed into a conductivimetric cell containing water. Conductivity changes versus time was plotted in order to determine the IT of the BO.

#### 3.5.4. Fourier Transforms Infrared Spectroscopy

BO without or with a high concentration of antioxidants (BO, BOAmx and BOTmx) were analyzed using a Prestige-21 FTIR spectrophotometer (Shimadzu Corporation, Kyoto, Japan) at 25 ± 2 °C. The samples stored at 60 ± 2 °C for 15 days were uniformly deposited in an attenuated total reflectance ZnSe crystal. The spectra were recorded from 4000 to 700 cm^−1^, with resolution of 4 cm^−1^, and 20 scans were accumulated. The data were analyzed in the IR solution software version 1.40 (Shimadzu Corporation, Kyoto, Japan).

### 3.6. Thermal Stability of Bullfrog Oil

Thermal stability was performed to obtain information on the physical and chemical changes of BOs using DSC/ TGA (STA 449F3, NETZSCH GmbH & Co, Selb, Germany). Samples (2 ± 0.5 mg) described in Section 3.5.1 were placed into aluminum pans. The parameters of analysis were: a heating rate of 10 °C·min^−1^, a synthetic airflow of 50 mL·min^−1^, and a temperature range of 25–600 °C. The isotherm of the bullfrog adipose tissue was performed using a DTG-60H (Shimadzu Corporation, Kyoto, Japan) at 70 ± 1 °C under a heating rate of 10 °C·min^−1^ and a flow of 50 mL·min^−1^.

### 3.7. Statistical Analyses

All results in this study were expressed as mean ± standard deviation of the analysis in triplicate. Statistical significance among groups was evaluated by variance analysis (ANOVA). Analyses between two groups were performed by a Student’s *t-*test. A value of *p* < 0.05 was considered statistically significant.

## 4. Conclusions

Physicochemical and chemical characterizations of bullfrog (*Rana catesbeiana* Shaw) oil were developed in order to produce oil quality parameters. The hot extraction method presented a high oil yield with low values of peroxide and acidity, and organoleptic characteristics without rancidity signals until the 60th day at room temperature. Compounds presented in the BO were efficiently identified by GC/MS analysis, exhibiting a composition rich in polyunsaturated fatty acids. The accelerated stability tests were efficiently applied to evaluate the quality of the BO. The techniques used were effective resources for monitoring primary and secondary products of oxidation and, therefore, oil degradation. Through the analytical methods used, it was observed that the lipid degradation of BO was time and temperature dependent, and characteristic of autoxidation processes. BHT was more effective for controlling BO oxidation, and exhibited a dose-response relationship. Although widely known and used as a synthetic antioxidant, BHT showed instability at high temperatures. Therefore, future research should test other groups of antioxidants such as the natural antioxidants tocopherols and ascorbic acid. This study also revealed that when used in approved quantities according to the International Excipient Guidelines and Regulations, BHT is safe and effective. In addition, the studied parameters were very helpful in evaluating the efficacy of the extraction and storage process, and to evaluate the thermal and oxidative stability of the oil. Therefore, BO appears to be a potential candidate for food, pharmaceutical and cosmetic applications, due to its rich chemical composition of mainly fatty acids, and its satisfactory thermal oxidative stability.

## Figures and Tables

**Table 1:** Chemical properties of extracted bullfrog oil. Composition (**A**) and oxidation induction time (**B**).

***A—****Percentage of major* *fatty acids from extracted* *bullfrog oil.* *compounds where composition was greater than 0.7% of total components. MW: molecular weight, RT (min): Retention time.			
Compound *	**MW**	**RT (min)**	**Area (%)**
Myristic acid (14:0)	300	10.35	1.4
Palmitoleic acid (16:1)	326	12.06	7.7
Palmitic acid (16:0)	328	12.25	10.3
Eicosapentanoic acid (20:5 *n*−3)	374	13.60	17.6
Oleic acid (18:1 *n*−9)	354	13.78	30.0
Stearic acid (18:0)	356	14.00	2.5
Arachidonic acid (20:4 *n*−6)	376	15.03	0.7
Docosahexaenoic acid (22:6 *n*−3)	400	16.51	0.8
Cholesterol	458	20.62	9.5
Ethyl-iso-allocholate	436	27.76	3.5
Total			83.9
Not identified			16.1
***B****—Oxidation induction time (IT) analyses of pure bullfrog oil (BO) performed at 110 °C and after addition of butylhydroxytoluene (BHA) or buthylhydroxyanisole (BHT) antioxidants at minimum (BOAm and BOTm) and maximum (BOAmx and BOTmx) concentrations.*			
**Sample**	**IT (h)**		
BO	0.32 ± 0.01		
BOAm	0.05 ± 0.01		
BOAmx	0.05 ± 0.03		
BOTm	0.72 ± 0.02		
BOTmx	7.88 ± 0.13		
